# Faces and Voices Processing in Human and Primate Brains: Rhythmic and Multimodal Mechanisms Underlying the Evolution and Development of Speech

**DOI:** 10.3389/fpsyg.2022.829083

**Published:** 2022-03-30

**Authors:** Maëva Michon, José Zamorano-Abramson, Francisco Aboitiz

**Affiliations:** ^1^Laboratory for Cognitive and Evolutionary Neuroscience, Department of Psychiatry, Faculty of Medicine, Interdisciplinary Center for Neuroscience, Pontificia Universidad Católica de Chile, Santiago, Chile; ^2^Centro de Estudios en Neurociencia Humana y Neuropsicología, Facultad de Psicología, Universidad Diego Portales, Santiago, Chile; ^3^Centro de Investigación en Complejidad Social, Facultad de Gobierno, Universidad del Desarrollo, Santiago, Chile

**Keywords:** visual speech, multimodal integration, imitation, primate social brain, speech evolution, speech development, audiovisual speech, face-voice integration

## Abstract

While influential works since the 1970s have widely assumed that imitation is an innate skill in both human and non-human primate neonates, recent empirical studies and meta-analyses have challenged this view, indicating other forms of reward-based learning as relevant factors in the development of social behavior. The visual input translation into matching motor output that underlies imitation abilities instead seems to develop along with social interactions and sensorimotor experience during infancy and childhood. Recently, a new visual stream has been identified in both human and non-human primate brains, updating the dual visual stream model. This third pathway is thought to be specialized for dynamics aspects of social perceptions such as eye-gaze, facial expression and crucially for audio-visual integration of speech. Here, we review empirical studies addressing an understudied but crucial aspect of speech and communication, namely the processing of visual orofacial cues (i.e., the perception of a speaker’s lips and tongue movements) and its integration with vocal auditory cues. Along this review, we offer new insights from our understanding of speech as the product of evolution and development of a rhythmic and multimodal organization of sensorimotor brain networks, supporting volitional motor control of the upper vocal tract and audio-visual voices-faces integration.

## Introduction

This review aims to integrate seemingly disparate evidence for different kinds of communicative behaviors (i.e., imitation, speech and lip-smacking) in humans and non-human primates (NHPs). Accounting for recently proposed anatomic-functional networks involved in primates’ social interactions, we attempt to provide new avenues for understanding how speech might have arisen from phylogenetically conserved multimodal and rhythmic neural properties.

We first address long-standing issues in the field of neonatal imitation research in both human and monkey newborns. In line with recent findings, we propose that rather than being exclusively innate, imitative behaviors are largely scaffolded by sensorimotor development and domain-general associative learning of multimodal information. Importantly, we argue that the development of these early abilities is largely supported by socially rewarding interactions with others. By the mean of these interactions, infants begin to associate what is seen (visual input), with what is heard (auditory input) and performed (motor output), and to learn the sensory consequences of their own and others’ actions. The evidence reviewed in section “Cross-Species Developmental Trajectories of Multimodal Integration” suggests that this socially guided and domain-general associative learning of multimodal information begins within the first year of life and could support the perceptual attunement for native auditory and visual speech. Once the perceptual system has narrowed in favor to the native stimuli present in their environment, infants can extract the regularities of their linguistic input and learn the multimodal associations between auditory (how it sounds), visual (how it is pronounced) and articulatory (how to pronounce it) aspects of their native language.

Then, we introduce the third visual pathway, a stream that was recently proposed to update the well-established model of the dual visual pathways and which is thought to be specialized for dynamic aspects of social perception. More specifically, the third visual pathway was shown to run laterally from V1 to the anterior temporal region along the superior temporal sulcus (STS) and to preferentially respond to biological movements of faces and bodies. The proponents of the third visual pathway report evidence supporting the involvement of STS in higher order social cognition, such as the recognition and understanding of others’ intentions and goals based on their actions and behaviors, including grasping movements, eye-gaze direction and facial expressions. Interestingly, the posterior portion of the STS is known to respond both to orofacial movements (i.e., speaking faces) and voices, making this region an ideal candidate to support the integration of faces and voices during audiovisual speech perception.

We begin the last section by reviewing the strongly reminiscent rhythmic pattern of human speech and monkey lip-smacking. Namely, these human and NHP communicative behaviors are highly rhythmic and produced at a particular rate within the theta frequency band. Remarkably, the synchronization of voices and mouth movements was documented not only during human speech production but also during monkey lip-smacking, where the acoustic envelop of vocalizations couples with inter-lips distance, both oscillating rhythmically around 4-to-5 Hz. This synchronization was recently documented in chimpanzees and marmoset monkeys, indicating that these coupled oscillations may have been crucial for the emergence of speech and must have evolved early in the primate lineage.

In section “Volitional Control of the Vocal Tract,” we emphasize on an important evolutionary adaptation of the structural connectivity of a cortico-subcortical network supporting the cognitive control of the vocal tract, which could have progressively allowed a finer control over speech sounds production. More specifically, the greater control over complex sequences of oral and vocal articulation that characterizes human speech compared to monkeys’ vocalizations could have been strengthened during evolution by more robust and direct connections between the laryngeal motor cortex and brainstem nuclei controlling volitional vocal folds vibrations as well as lips and tongue movements.

Finally, we report evidence of cross-species similarities and differences in developmental trajectories for audiovisual speech perception. Namely, during the first year of life infants show a progressive specialization of auditory (phonemes, vocalizations) and visual (faces, speaking mouths) systems for the discrimination of native input, at the cost of non-native input. This developmental pattern is known as “perceptual narrowing” and has been described in both human and NHP infants with analogous timing. Interestingly however, although human and monkey infants exhibit a similar interest for the eyes, monkeys’ infants have been shown to pay less attention to the mouths, a region of other’s faces that convey critical visual communicative cues that facilitate the auditory processing of communicative vocal behaviors and foster expressive language development.

## Homo Imitans? Methodological and Theoretical Controversies

### Do Humans Imitate From Birth?

**FIGURE 1:**
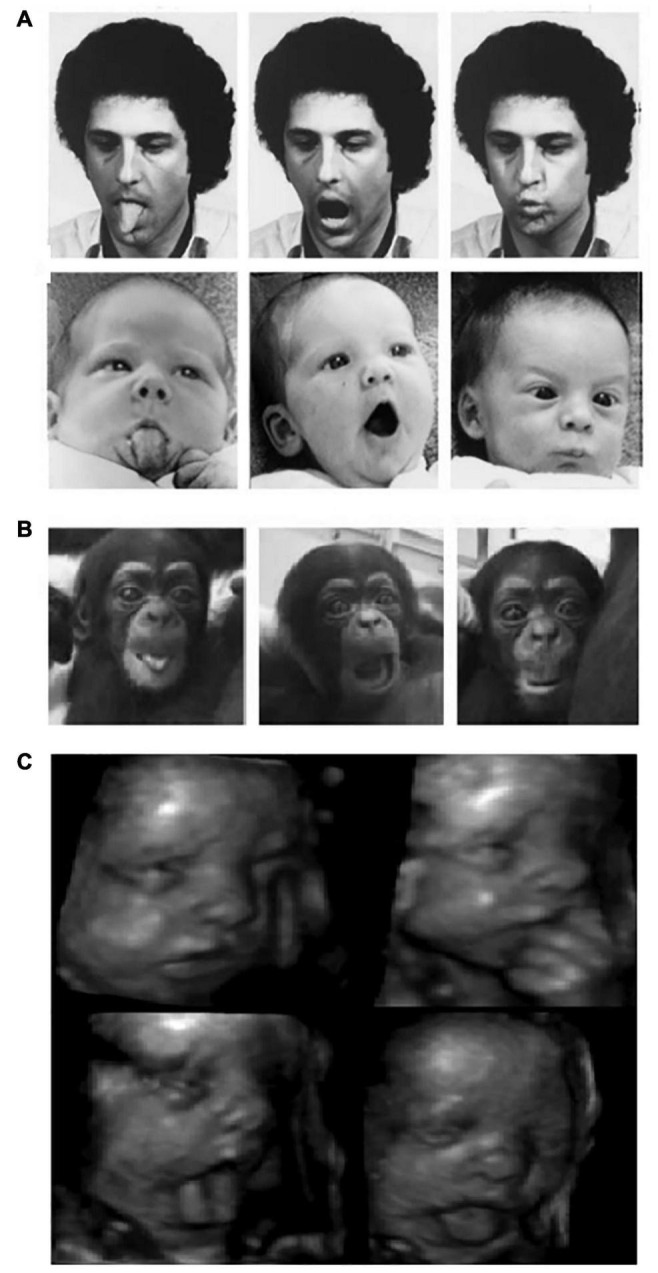
**(A)** Human and **(B)** chimpanzee neonates imitating orofacial gestures (left panel: tongue protrusion; middle panel: mouth-opening; right panel: lips protrusion) [**(A)** Reprinted with permission from Meltzoff and Moore (1977) and **(B)** Reprinted with permission from Myowa-Yamakoshi et al. (2004)]. **(C)** A twenty-eight-week gestational age fetus producing aerodigestive stereotypies Reprinted with permission from Kurjak et al. (2004).

In psychological science, imitation is understood as the ability to copy the topography of a behavior (e.g., body movements, vocal or facial expressions) observed in a third person or agent (Heyes, 2021). However, researchers distinguish several forms of imitation that may differ in the complexity of their cognitive underpinnings (Zentall, 2012). An accurate imitation requires the imitator to generate a correspondence between what is seen or heard and what is performed. In other words, crossmodal associations are needed to map the visual or auditory information provided by the model into a matching motor sequence. The main problem raised by imitation is how these sensorimotor associations are established and by means of which neurocognitive mechanisms. This problem is known as the “correspondence problem” and it is still vividly debated in the scientific community. Since the late 1970s, influential works have argued that the ability to imitate is already present in neonates from 2-to-3 weeks old who successfully imitate facial gestures such as tongue or lip protrusion and mouth-opening (see Figure 1A; Meltzoff and Moore, 1977, 1997; Meltzoff, 1988). These results led to introduce the popular idea of an innate, hardwired module for imitation and human infants started to be considered as “Homo imitans” (Meltzoff, 1988).

Although debated for several decades, it was not until recently that neonate imitation became one of the most controversial phenomena in the field of developmental cognitive science (Kennedy-Costantini et al., 2017; Heyes et al., 2020; Davis et al., 2021). The skepticism around the idea that imitation is in our genes arose with several studies showing that neonates elicit facial gestures in response to different kind of stimuli (Jones, 2017; Keven and Akins, 2017). For example, 4-week-old infants were as likely to elicit tongue protrusion when listening to music or seeing flashlights as when observing a model performing tongue protrusion (Jones, 1996, 2006), suggesting that the production of such gestures are not specifically intended to be imitative behaviors. More crucially, a recent longitudinal study involving more than 100 newborns failed to find evidence of imitation for any of the 9 action-types tested at 1, 3, 6 and 9 weeks of life using the same method as the inaugural works of the 1970s (Oostenbroek et al., 2016). This year, a meta-analysis of 336 effect sizes (Davis et al., 2021) shed serious doubts on the reliability of the evidence supporting the notion of Homo imitans. They demonstrated that the results of neonatal imitation research present an important heterogeneity that cannot be explained by methodological factors but is rather modulated by a “researcher affiliation” effect, with some laboratories being more likely to report larger effects. Finally, it is a possibility that a publication bias in the field may have increased the propensity for positive results to get published and negative ones unpublished (Ferguson and Heene, 2012; Heyes, 2016; Slaughter, 2021).

### Do Non-human Primate Neonates Imitate?

Whether or not the neonatal imitation abilities observed in human infants are present in NHPs has been particularly challenging to evidence with robust results. A study conducted on two chimpanzee neonates younger than a week of age revealed that they were able to imitate different types of human orofacial gestures (see Figure 1B). The authors claimed that, because of their very young age, the chimpanzees had very few opportunities for learning visuomotor associations, suggesting that they “are born with the ability to match visually perceived oral gestures with a proprioceptive motor scheme” (Myowa-Yamakoshi et al., 2004). Similarly, Ferrari et al. (2006) tested a group of 21 infant rhesus macaques at the age of 1, 3, 7 and 14 days and reported imitative behaviors for 2/6 of the actions tested, namely lip-smacking and tongue protrusion. It is noteworthy, however, that these two oral gestures were imitated only at 3 days of age, nor earlier nor later (Ferrari et al., 2006).

Nearly around the same time when the concept of Homo imitans began to be severely questioned, a study performing a re-analysis of data for neonatal imitation in rhesus macaques revealed no supporting evidence. Redshaw (2019) claimed that the gold standard cross-target approach, which controls that gestures are exhibited specifically in response to the same modeled action, is not correctly implemented in most the studies of the phenomenon. Importantly, he re-analyzed the dataset of the 163 individuals ever tested to date using cross-target analysis and demonstrated that correct matching tongue protrusion and lip-smacking responses in macaque neonates were not produced at levels greater than chance (Redshaw, 2019). For instance, lip-smacking was produced at the same odds in response to observed lip-smacking and mouth-opening. Similarly to the unspecific human neonates’ tongue-protrusion behaviors in response to the same action, to music or flashlights, this study rules out the possibility that such gestures are actually imitative. Although the debate is far from being solved (Meltzoff et al., 2018, 2019; Oostenbroek et al., 2018), the controversy at the heart of the field has strongly challenged the existence of neonatal imitation abilities in both human and NHPs.

### In-Born Module for Imitation or Sensorimotor Development?

Similar developmental trajectories of imitation were documented for humans and chimpanzees. Several studies have shown that tongue protrusion imitation observed during the first few weeks after birth in both species progressively disappear around the end of the second month of life (Abravanel and Sigafoos, 1984; Myowa-Yamakoshi et al., 2004; Subiaul, 2010; Jones, 2017). Some authors advocating for neonatal imitation explain that this decrease in the incidence of orofacial imitation is “probably due to the maturation of the cortical mechanisms inhibiting unwanted movements that follows the development of the organization of motor control […] and reappears at an older age in terms of intentional imitation” (Rizzolatti and Fogassi, 2016, p.382). Although it is unclear whether imitation is present from birth, it is undeniable that this faculty develops within the first years of life. An alternative explanation we are more inclined to, formulated by detractors of neonatal imitation, propose that imitative behaviors require sensorimotor learning which instead start to emerge at the end of the first year and extend over infancy and childhood (Jones, 2017; Slaughter, 2021).

In a recent article that received more than 20 peer commentaries (most of which agreed that evidence for neonatal imitation is unreliable), Keven and Akins (2017) proposed that the orofacial gestures observed in neonatal imitation research, specifically tongue protrusion and mouth opening, are in fact motor stereotypies associated with perinatal aerodigestive development in mammalians. These stereotypies begin during gestation and last until respiratory and swallowing systems begin to prepare for the introduction of solid food, around month 3. As depicted in Figure 1C, ultrasound images of fetuses have shown that a variety of the orofacial gestures discussed above are already consolidated at approximately 28 weeks of gestational age (De Vries et al., 1984; D’Elia et al., 2001; Hata et al., 2005). Since these gestures are spontaneously produced both in the womb (without any model) and perinatal life but disappear around 3 months, neonatal imitation could represent an epiphenomenon better explained by sensorimotor development. Crucially for the purpose of the current review, Keven and Akins (2017) also proposed that perinatal stereotypic gestures participate in the acquisition of orofacial motor control that, in turn, may support not only swallowing of solid food but also motor biomechanics for speech-like sounds production emerging by month 3 (also see Choi et al., 2017; Mayer et al., 2017).

### Imitation, Mirror Neurons and Communication

An increasing number of studies using causal (transcranial magnetic stimulation; TMS) and lesion methodologies demonstrate that brain areas typically displaying mirror properties are involved in imitation. It has been shown that inhibitory repetitive TMS of the inferior frontal gyrus (IFG) specifically impairs imitative behaviors (Heiser et al., 2003; Catmur et al., 2009) and that excitatory stimulation of the same area improves vocal imitation (Restle et al., 2012). Other mirror neuron areas of the precentral gyrus and inferior parietal region are thought to be implicated as well (Binder et al., 2017; Reader et al., 2018). Similar to the debated innateness of imitation, the origins of mirror neurons have been the object of an intense nature vs. nurture debate. Importantly, the proponents of the mirror neuron theory take neonatal imitation as evidence for the presence of mirror properties from birth and suggest that they are part of an innate system for action-perception (Simpson et al., 2014). On the other hand, accordingly to those who defend that imitation emerges later during infancy, “neurons acquire their mirror properties through sensorimotor learning” (Heyes and Catmur, 2022). Mirror neurons were originally observed when visuomotor neurons in the monkey premotor cortex began to fire not only when a monkey executed a grasping task but also when it observed the researcher performing this grasping behavior (di Pellegrino et al., 1992). While for methodological reasons in humans there is little direct evidence for mirror neurons, a mirror system has been proposed to be involved in the simulation of others’ behaviors, providing a “view from the inside” of the observed conduct (Rizzolatti and Craighero, 2004; Rizzolatti and Sinigaglia, 2008). After these findings, mirror neurons were proposed by some authors to represent the neural mechanism involved in imitation skills (Cross et al., 2009; Iacoboni, 2009). Nonetheless, it remains unclear whether mirror neurons emerge from some modular, inherited mechanism where the others’ behavior is somehow represented in the mirror neuron system, or whether they result from domain-general processes like associative learning. One view is that grasping mirror neurons participate in hand visuomotor control, which by associative mechanisms may extend to the observation of others beside the own hand (Oztop and Arbib, 2002; Kilner et al., 2007). Once their function has been amplified to the observation of others’ behaviors beside the own, the motor programs become modulated by the former resulting in progressive imitation. As opposed to the representational view, this perspective provides a mechanistic interpretation of the mirror neuron mechanisms based on known processes of neuronal plasticity and development (Aboitiz, 2017, 2018b).

Mirror neurons have also been proposed to play an important role for communication and social cognition in both humans and NHPs. Specular activity between interacting individuals is thought to be a mechanism contributing to the formation of social bonds, especially between caregivers and their offsprings. Mother-child dyads observation, for instance, revealed that mothers actually imitate their infants’ facial gestures and vocalizations to a greater extent than infants imitate their parents (Jones, 2006; Athari et al., 2021). Parental imitative behaviors offer a form of reward-based learning for infants that may reinforce the elaboration of early learned associations between the self-generated motor sequences and the resulting perceptual outcomes—visual outcomes for imitative facial gestures but also auditory outcomes for vocal imitation—in the other person. Crucially, until they are exposed to real mirrors, infants have no visual feedback over their own face when gesturing (unlike for their arms and legs movements) and therefore, could use caregivers’ imitations as “social mirrors” to gain knowledge into crossmodal mapping (Ray and Heyes, 2011).

In sum and based on the evidence revised above, we argue that imitation as well as speech are social abilities that develop during infancy alongside with sensorimotor systems and require associative learning of multimodal input. The purpose of the following sections of this review is to emphasize on the importance of these crossmodal associations between what is performed, what is seen and what is heard (motor-visual-auditory) for the evolution and development of human speech.

## A Brain Network for Dynamic Faces and Voices Perception

### A Third Visual Pathway?

Forty years ago, Ungerleider and Mishkin (1982) evidenced that the primate visual cortex is organized in two streams. A decade later, Goodale and Milner (1992) demonstrated a similar dual organization in the human brain, with a dorsal and ventral pathway distinguishable both anatomically and functionally. The dorsal stream also known as the “where and how” stream, projects from early visual cortices and reaches the prefrontal cortex running along the parietal lobe. This stream was proposed to underly the processing of visual information about objects’ spatial location and the execution of actions related to these objects. The ventral stream, also known as the “what” stream, runs from early visual cortices toward the inferior temporal lobe and is widely thought to support object identification (e.g., animals, cars, faces). The two-visual pathways model has not only been one of the most influential models for visual system organization in the brain, but it has also influenced important models of auditory cortical processing (Kaas and Hackett, 1999; Romanski et al., 1999; Romanski, 2007), attentional networks (Corbetta and Shulman, 2002) and the neurobiology of language (Hickok and Poeppel, 2004, 2007) in which dorsal and ventral streams are described accordingly to their “where and how” and “what” functions, respectively. In the particular case of language processing in the brain, the dorsal pathway is proposed to connect posterior superior regions of the temporal lobe with the frontal cortex, allowing the mapping of speech sounds with the orofacial articulatory sequences required to produce these sounds. The ventral pathway, connecting posterior to anterior areas of the middle and inferior temporal gyri, is believed to support the mapping of speech sounds onto linguistic meaning (Hickok and Poeppel, 2004).

**FIGURE 2:**
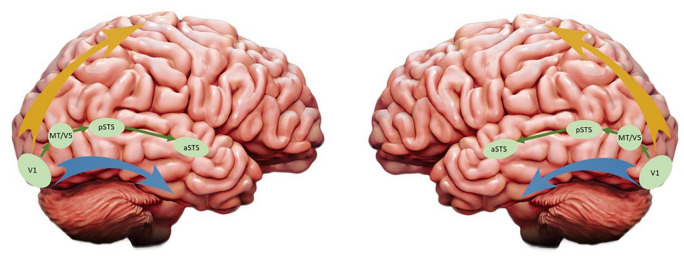
Updated version of the visual streams model: The ventral and dorsal pathways are represented by the blue and yellow arrays, respectively, and the third visual pathway proposed by Pitcher and Ungerleider (2020) is depicted in green. While these authors emphasized the role of the third pathway in the right hemisphere (left panel), in this article we are focusing on its functions in the left hemisphere (right panel).

Last year Leslie G. Ungerleider, who first reported the dual organization of visual processing in primates’ cortex (Ungerleider and Mishkin, 1982), and David Pitcher reported compelling evidence for the existence of a third visual pathway and claimed that the two-visual pathways model needs to be updated (Pitcher and Ungerleider, 2020). Reviewing evidence coming from fMRI, TMS, lesion, tracers and tractography studies, they proposed that this third visual pathway is anatomically and functionally segregated from the existing dorsal and ventral streams, projecting on the lateral part of both human and NHP brains and specialized for social perception. Originating in the primary visual cortex (V1), the third pathway sends projections into the posterior and anterior portions of the superior temporal sulcus (pSTS and aSTS, respectively) *via* the area V5/MT (see Figure 2), an area well known for its responsiveness to visual motion. In both monkeys and humans, the aSTS displays selective responses to moving but not to static faces and bodies (Zhang et al., 2020), a functional characteristic that differs from those face areas of the ventral stream (which include the occipital and fusiform face areas for a more static and structural identification of faces). Altogether the evidence reported by the authors emphasizes the role of this lateral pathway in the processing of a wide range of socially relevant visual cues and, by extension, in higher order social perception. For instance, based on the eye-gaze direction or hand movements of our interlocutors, humans are able to generate predictions about their goals and intentions. In other words, the existence of a third visual pathway specialized for the perception of facial and corporal dynamics may have supported the human brain readiness for social interactions.

### A Possible Function for the Third Visual Pathway in the Left Hemisphere

Although a great emphasis was made on the right STS, the authors were more elusive with respect to the role of the third visual pathway in the left hemisphere. In fact, they leave the following questions open: “Is the third pathway lateralized to the right hemisphere in humans? If so, what are the visual functions of the left STS and what is the role of speech?” (Pitcher and Ungerleider, 2020). Here, we advocate for the existence of a third visual pathway for social perception in the left hemisphere and review evidence of the special role of STS for the evolution of multimodal integration of speech.

Decades of research on the STS have consistently demonstrated that it supports the audiovisual integration of faces and voices. Neuronal populations of the macaque STS have been shown to respond to both auditory and visual stimuli, especially when the heard vocalizations matched the seen mouth movements. Interestingly, this pattern of responses for face/voice perception has been observed in the right (Perrodin et al., 2014) and the left hemisphere (Ghazanfar et al., 2005, 2008). More recently, in a study using single neuron recordings of face patches in macaques’ left (*n* = 3) and right (*n* = 1) hemisphere, Khandhadia et al. (2021) reported greater responses to audiovisual stimuli in the face patch AF (in the aSTS) with respect to AM (in the undersurface of the temporal lobe). These results are consistent with the functional distinction between a lateral visual pathway specialized in social perception of moving faces and a ventral pathway dedicated to more static, structural and unimodal aspects of face processing. In humans, both right and left STS have been reported to process communicative facial and vocal cues, with preferential responses to audiovisual face-voice stimuli and no responses to manual gestures (Deen et al., 2020). Other fMRI studies have reported that different areas of the pSTS are responsive to mouth and eye movements (Puce et al., 1998). Interestingly, only the anterior portion that prefers mouth-movements elicited strong responses to voices, contrasting with the posterior portion who responded to eye-movements but not to voices (Zhu and Beauchamp, 2017; Rennig and Beauchamp, 2018). The latter suggests that vocal sounds and the orofacial movements that produce them are integrated in the anterior pSTS. In line with this functional specialization, a recent study reported homologous representation of conspecific vocalizations in bilateral auditory cortices of humans and macaques. More specifically, this temporal voice area is located in the anterior temporal lobe, dorsally to STS (Bodin et al., 2021).

It is noteworthy that, before the third visual pathway for social perception was formally proposed, neurobiological models of audiovisual speech processing already had included the left MT/V5 and pSTS as critical areas (Bernstein and Liebenthal, 2014; Beauchamp, 2016; Hickok et al., 2018). Additionally, the STS has been proposed to be critical for semantic processing, serving as an interface between the auditory component of speech perception and the visual recognition system, providing a substrate for the representation of content words and scenes containing schemas of agents and objects (Aboitiz, 2018a).

## Evolution and Development of Multimodal Integration in the Primate Brain

### The Rhythmic Evolution of Communication: From Lip-Smacking to Human-Speech Rhythm

Speech is produced rhythmically and its temporal structure remains stable across languages, within the range of 2-to-7 Hz with a notable peak in the theta frequency band between 4 and 5 Hz (Poeppel and Assaneo, 2020). Interestingly, the spectral frequency of the speech envelope corresponds to the rate of syllable production (Park et al., 2016). In turn, the acoustic envelopes of speech and orofacial speech movements seem to be tightly time-locked, both modulated in the 2-to-7 Hz frequency range. Chandrasekaran et al. (2009) have measured and correlated the speech envelope with the area of mouth opening associated to spontaneous production in English and French audiovisual speech datasets. Their analysis revealed robust correlations between inter-lip distance and speech sounds amplitude but also a consistent interval of 100-to-300 ms between the onset of visual speech (the initial, visible lip movements) and the onset of the corresponding speech sound. This mouth/voice orchestration suggests that, before the brain proceeds with multimodal speech processing, stable and redundant temporal information are already embedded in the audiovisual speech stream itself (Chandrasekaran et al., 2009).

**FIGURE 3:**
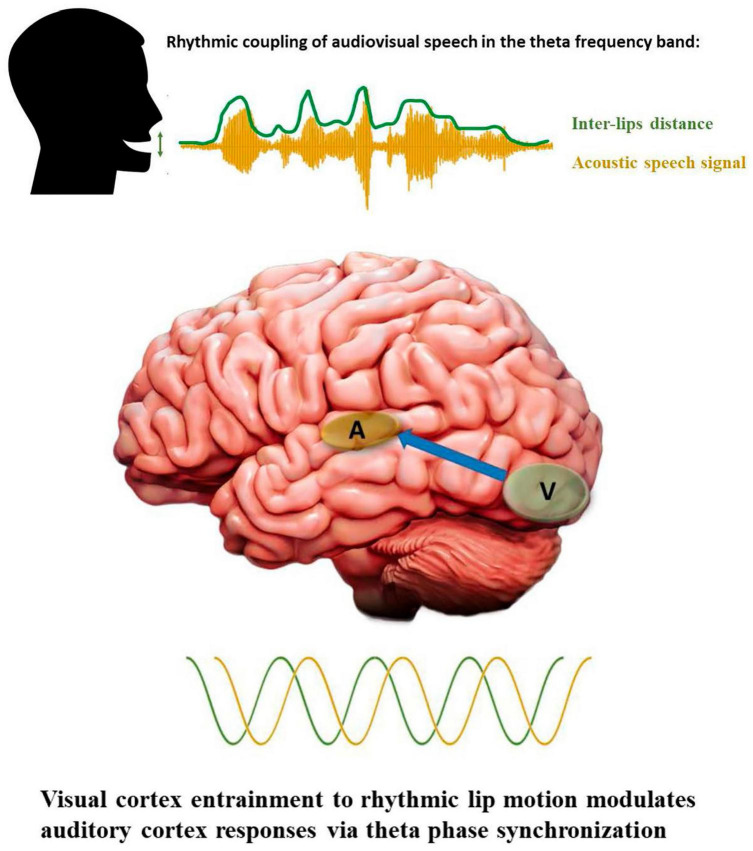
Rhythmic properties of audiovisual speech and cortical oscillations. Visual and auditory speech cues as well as neural oscillations are depicted in green and yellow, respectively. The blue array in the bottom panel indicates feedforward modulation of auditory cortex responses *via* theta synchronization to visual cortex oscillatory rhythm.

During face-to-face conversations, humans take advantage of visual information provided by the speaker’s mouth movements to facilitate speech comprehension, especially when the surrounding environment is noisy (Sumby and Pollack, 1954; also see Crosse et al., 2015). Recent studies have begun to uncover the underlying mechanisms of audiovisual integration in the human brain. Electrophysiological recordings have reported that visual speech speeds up the processing of auditory speech (Van Wassenhove et al., 2005) and allows crossmodal predictions (Michon et al., 2020). This temporal facilitation is consistently reflected by shorter latencies and lower amplitudes of the auditory components N1 and P2 [see Baart (2016) for a critical review]. Interestingly, the facilitation effect and crossmodal predictions are more pronounced for those visual speech cues with salient places of articulation in the upper vocal tract (e.g., bilabial consonant-vowel/ba/) with respect to those produced in the lower vocal tract which are visually less salient (e.g., velar consonant-vowel/ga/). The analysis of oscillatory brain activity has also offered critical insights with respect to audiovisual integration and crossmodal predictions. Using magnetoencephalography, Park and collaborators demonstrated that the perception of speaking lips entrains visual cortex oscillations and modulates the activity of the auditory cortex (Park et al., 2016). In line with these results, a recent study using intracortical recordings reported that neurons of the auditory cortex track the temporal dynamics of visual speech cues based on their phase of oscillations (Mégevand et al., 2020). Another intracortical study found a sub-additive effect in which responses to audiovisual speech were weaker compared to auditory speech only in the left posterior superior temporal gyrus, suggesting that visual speech optimizes auditory processing efficiency (Metzger et al., 2020). Importantly, a partial coherence between the left motor region oscillations and lip movements rate have also been identified that directly predicted the participants performance on comprehension, suggesting that motor cortex could facilitate the integration of audiovisual speech through predictive coding and active sensing (Park et al., 2016, 2018). Several recent studies have proposed that visual cortex entrainment to rhythmic lip motion modulates the responses of auditory cortex *via* theta phase synchronization (Crosse et al., 2015; Zoefel, 2021; see Figure 3), including when visual speech only is presented (Bourguignon et al., 2020; Biau et al., 2021).

Human speech is rhythmic and multimodal; our voices and mouth movements are temporally coordinated when we speak and the oscillatory activity of our brain couples with and exploits the statistical regularities present in the audiovisual input to improve speech perception/comprehension (Figure 3). There is now a growing body of studies revealing a similar temporal structure is present in NHP communication. Primates’ vocalizations and communicative calls have been shown to synchronize with the rhythm of facial expressions, such as mouth opening/closing during lip-smacking behaviors. This synchronization between vocalizations and lips movements has been reported in marmosets, macaque rhesus monkeys and chimpanzees. Critically for evolutionary accounts of audiovisual speech perception, it appears to be phase-locked in the theta band frequency, matching the syllable production rate observed in humans at approximately 4 Hz (Ghazanfar et al., 2013; Ghazanfar and Takahashi, 2014a,b; Gustison and Bergman, 2017; Pereira et al., 2020; Risueno-Segovia and Hage, 2020). The NHP brain is highly tuned to facial expressions accompanying affiliative calls and, similar to humans, take advantage of orofacial visual cues to speed up auditory processing and to enhance the perception of vocalizations in noisy environments (Chandrasekaran et al., 2011, 2013). Interestingly, the neural mechanisms underlying these behavioral advantages seem to be similar across species, reflected by reduced or suppressed responses in auditory neurons for multimodal compared to unimodal auditory perception (Ghazanfar and Lemus, 2010; Kayser et al., 2010).

Altogether the evidence reviewed above demonstrates that both human and NHPs communicate rhythmically, producing coordinated vocalizations and orofacial gestures around 4–5 Hz. Their neural oscillations synchronize to this frequency and take benefit from the consistency of audiovisual regularities in voice onset and mouth opening co-occurrence. Noticeably, the syllable production rate observed across all human languages is already present in the marmoset lip-smacking, suggesting that rhythmic communication may have evolved early in the primate lineage.

### Volitional Control of the Vocal Tract

Additionally to their analogous rhythmic patterns, the production of human speech and primate lip-smacking involves a common cortical network including the IFG, the ventrolateral and dorsomedial prefrontal cortex (vlPFC and dmPFC) in humans and NHPs (Rizzolatti and Craighero, 2004; Petrides, 2005; García et al., 2014; Neubert et al., 2014). These shared anatomico-functional properties across species are in line with previous cytoarchitectonic studies establishing the vlPFC as the NHP homolog of Broca’s area, both structures being responsible for the initiation of vocal communicative behaviors (Petrides and Pandya, 2002; Petrides et al., 2005). In macaques, cognitive control required to produce volitional vocalizations has been shown to consistently recruit the IFG (Gavrilov et al., 2017; Loh et al., 2017; Shepherd and Freiwald, 2018). Other studies, using single neuron recordings, confirmed that the vlPFC elicits dedicated responses during volitional initiation of vocalizations (Hage and Nieder, 2013; Gavrilov and Nieder, 2021). Recent research in humans indicates that left vlPFC and premotor cortex also supports the control of voluntary orofacial movements (Loh et al., 2020; Maffei et al., 2020). This evidence suggests that the inferior frontal region has an ancestral role for orofacial (lip-smacking) and vocal (affiliative calls) control in NHP communication, which could be regarded as a phylogenetic precursor of human speech control. Because human vocalizations are much more complex, it was argued for decades that primate lip-smacking and orofacial communication could not have served as an evolutionary building block of human speech. More recently however, accounting for the above-mentioned evidence, emerging theories are advocating for a common evolutionary origin of vocal-facial communicative gestures that could have arisen well before the hominin radiation (Aboitiz and García, 1997; Morrill et al., 2012; Ghazanfar, 2013; Ghazanfar and Takahashi, 2014a,b; Shepherd and Freiwald, 2018; Michon et al., 2019; Brown et al., 2021).

Importantly, the phylogenetic role of vlPFC is not limited to the control of orofacial effectors for the production of speech and communicative behaviors but extends to perception as well. In humans as in NHPs, the visual and auditory ventral pathways project axonal terminals into vlPFC (Romanski, 2007; Hage and Nieder, 2016). In line with this structural overlap, a neural population was found in the vlPFC of rhesus monkeys that responds to the perception of both conspecific faces and vocalizations (Sugihara et al., 2006; Romanski, 2012; Diehl and Romanski, 2013) and is also recruited when monkeys produced vocalizations (Hage and Nieder, 2015). Moreover, a recent study using electric stimulation combined with fMRI revealed a common effective connectivity between auditory cortex and vlPFC in human and monkey brains (Rocchi et al., 2021). These results turn the vlPFC into a phylogenetically conserved trimodal region for the integration of audiovisual and motoric aspects of communication that may have contributed to the emergence of human speech (Michon et al., 2019).

It is noteworthy that the synchronization of speaking mouths and voices around the 4.5 Hz has been proposed to emerge as a consequence of an intrinsic speech-motor rhythm observed in humans (Assaneo and Poeppel, 2018). In other words, mouth movements and vocalizations couple around the same frequency band because they both represent the sensory consequences of complex sequences of the orofacial effectors and vocal tract movements, which are produced at this particular rhythm. Using principal component analysis to investigate the joint variation of facial and vocal movements, a recent study combining videos of human faces articulating speech and MRI sequences of the speaker’s vocal tract has shown that sufficient information is available in the configuration of a speaking face to recover the full configuration of the vocal tract (Scholes et al., 2020). The part of the face that contributes the most to the recovery of vocal tract configuration are those parts who are required to produce speech sounds (e.g., upper and lower lips for bilabial phonemes or the back of the tongue for velar phonemes). In humans, the LMC is thought to be located in the primary motor cortex, more specifically in area 4 and to have direct monosynaptic projections to the ambiguous nucleus, the seat of laryngeal motoneurons in the brainstem controlling the vibration of the vocal cords. In NHPs by contrast, the LMC is located in the area 6 of the premotor cortex and connects to laryngeal motoneurons only indirectly *via* interneurons of the reticular formation (Simonyan and Horwitz, 2011; Simonyan, 2014). Additionally, tractography analyses have revealed that human LMC connectivity with somatosensory and inferior parietal cortices are strongly enhanced compared to its NHP homolog (Kumar et al., 2016). The latter suggests that the evolution of LMC connectivity with both brainstem nuclei and temporoparietal cortex may have contributed to a greater control over the vocal tract for volitional vocalizations and to higher-order sensorimotor coordination in response to social perception demands, respectively. Recently, both anatomic and functional research have proposed a division of the human LMC into a dorsal and a ventral portion (Belyk et al., 2021; Hickok et al., 2021). The dorsal laryngeal motor cortex (dLMC) has been shown to be causally involved in the control of laryngeal muscles involved in voluntary vocalizations and vocal pitch modulations used to convey meaning in human speech production (Dichter et al., 2018). The dLMC shows greater connectivity and a consistent role in laryngeal motor control whereas the ventral one has fewer projections, suggesting that it could be part of the premotor cortex as NHPs’ LMC (Dichter et al., 2018; Eichert et al., 2020). Even though it was recently associated with verbal fluency in individuals who stutter (Neef et al., 2021) and with respiration coordination for vocal-motor control (Belyk et al., 2021), the function of the ventral LMC remains mostly unknown.

In sum, the evidence reviewed in this section indicates that humans and NHPs present structural and functional homologies for the volitional control of the vocal tract in the vlPFC. Crucially, in addition to its role in vocal production, this region also responds to the perception of vocalizations and orofacial movements in both species. According to its phylogenetically conserved anatomical and functional features, we argue that the vlPFC plays a critical role in the integration of audiovisual and motoric aspects of communication and may have contributed to the emergence of human speech. Nevertheless, important cross-species differences have been documented in the connectivity between LMC and brainstem nuclei, specifically the connections to the ambiguous nucleus are more robust and direct in human brains compared to NHP brains. This difference of connectivity strength could explain why human speech has evolved toward more complex vocal and orofacial sequences compared to NHP lip-smacking (Brown et al., 2021).

### Cross-Species Developmental Trajectories of Multimodal Integration

One of the first multimodal associations that an infant must learn is the matching between her caregivers’ faces and voices. During their first months of life, human infants are capable to discriminate a wide variety of non-native stimuli but loose this ability by the end of the first year. This counterintuitive developmental pattern of perception is known as perceptual narrowing and has been described for speech sounds, faces (Kuhl et al., 2006; Krasotkina et al., 2021) and music (Hannon and Trehub, 2005). For instance, 6-to-8 but not 10-to-12 months old English infants were capable to discriminate non-native phonemic contrasts (Werker and Tees, 1984). At a similar developmental timing, the same phenomenon occurs for non-native faces, including faces from different races (Kelly et al., 2007) or species (Pascalis et al., 2002). Interestingly, the visual discrimination of speech is also subject to a perceptual narrowing between 6 and 11 months of age (Pons et al., 2009). An accepted interpretation of this regression in the perception of non-native stimuli propose that the visual and auditory systems are progressively tuning in favor of the particular input infants are exposed to (i.e., native faces and speech sounds). The refinement of perception for conspecific’s voices and faces is thought to optimize the processing of the relevant information used within one’s native social group (Lewkowicz and Ghazanfar, 2009).

As mentioned above, monkey lip-smacking and human speech converge on a ∼5 Hz rhythm but they were also demonstrated to share homologous developmental mechanisms strongly supporting “the idea that human speech rhythm evolved from the rhythmic facial expressions of our primate ancestors” (Morrill et al., 2012, p.3). In both NHPs and humans, environmental variables seem to foster the development of social perception skills. Dahl et al. (2013) investigated the development of face perception in a colony of captive young and older chimpanzees with lifelong exposure to non-conspecific faces (human scientists) and showed that younger apes discriminate conspecific faces better than human faces, but older apes elicited the opposite pattern, discriminating better human than conspecific faces. The results suggest the existence of early mechanisms that favor perception tuning toward native-species stimuli and of late mechanisms that narrow the perceptual system along with the critical information of the faces frequently encountered in the environment (for older captive monkeys, human faces). Controlling for genetics, perinatal experience and growth, a study conducted on infant marmoset twins who were exposed to different amount of social reinforcement demonstrated that infants receiving more contingent parental feedback show an increased rate of vocal development with respect to their twins who were provided less contingent feedback (Takahashi et al., 2017). Another example of the role of experience are human infants raised in bilingual environment, who exhibit a prolonged perceptual narrowing (Werker and Hensch, 2015). Bilingually raised infants were able to discriminate non-native speech sounds, which age-matched monolingual infants were no longer able to discriminate (Petitto et al., 2012; Byers-Heinlein and Fennell, 2014; also see Kuhl et al., 2003). This influence of linguistic exposure has also been reported for visual discrimination of speech (Weikum et al., 2007; Sebastián-Gallés et al., 2012).

It is known that around the sixth month, when infants start babbling, they start to spend more time looking at the part of the face that conveys linguistic information (i.e., the mouth) and that visual attention returns to the eyes around the end of the first year when they have formed their native phonological repertoire (Lewkowicz and Hansen-Tift, 2012). Bilingual infants attend more to the mouth than to the eyes of a speaking face from an earlier age and for a longer period of time, taking advantage of the multimodal input to support the acquisition of their two languages and respective phonological repertoires (Pons et al., 2015). It is noteworthy that the additional linguistic information provided by lip movements has recently been demonstrated to foster expressive language skills during the second half of the first year (Tsang et al., 2018) and improve the learning and recognition of novel words in 24 months-old monolinguals and bilingual toddlers (Weatherhead et al., 2021). Interestingly, this preferential orientation of visual attention toward the mouth has been reported in adults as well; when exposed to their second non-native language, adults attend more to the speaking mouth independently of their level of proficiency (Birulés et al., 2020). Adjusting for between species difference in developmental timescale, a recent study compared infant rhesus macaques’ and human infants’ face processing strategies revealing a highly similar U-shape pattern of changes in visual engagement with the eyes of unfamiliar conspecifics. However, they also showed that human infants visually engage with the mouth to a greater extent than macaque infants do, suggesting that the process of language acquisition may require an increased reliance on the information conveyed by orofacial movements (Wang et al., 2020). Using functional near-infrared spectroscopy, Altvater-Mackensen and Grossmann (2016) reported that 6-month-old infants who prefer to look at speakers’ mouths exhibit enhanced responses in the left inferior frontal cortex compared to those infants who prefer the eyes of a speaker. Accordingly with the functions of the IFG discussed above (see section “Volitional Control of the Vocal Tract”), the authors conclude that this region plays a crucial role for multimodal association during native language attunement (Altvater-Mackensen and Grossmann, 2016).

Taken together the evidence supports the idea that, despite some differences of rate due to their heterochronous neural development, humans and NHPs share similar developmental trajectories for multimodal integration of social stimuli. Noticeably, within their first year of life, infants of both species show a progressive attunement for the processing of native or species-specific visual (faces) and auditory (vocalizations) social stimuli.

## Discussion

The current review addresses the rhythmic and multimodal aspects of communication and brain mechanisms that could have scaffolded human brain readiness for social interactions during evolution. Particular emphasis was placed on the importance of sensorimotor development, in domain-general associative learning of multimodal information and in socially rewarding interactions for the development of communicative behavior like imitation and speech during infancy. On the other hand, we integrate recent evidence of anatomical and functional homologies and differences between humans’ and non-human primates’ social brain, specifically for the perceptual processing of dynamic social cues (such as voices and faces) and for the volitional control of the vocal tract. We propose to synthesize the findings of this review around 5 questions that, in our view, contribute to better understand the domain-general mechanisms and properties of the primate brain underlying the evolution and development of speech.

### In-Born Module for Imitation or Sensorimotor Development?

We began this review by addressing the controversies surrounding the longstanding theory of neonatal imitation in humans and NHPs. Recent data re-analysis and meta-analysis have raised serious issues concerning the reliability of the gold-standard methods used in neonatal imitation research. As a consequence, the idea of Homo imitans with innate imitative abilities has been strongly challenged. Alternatively, imitation may rely on crossmodal associations of sensorimotor information (e.g., visuomotor associations for facial imitation and audiomotor associations for vocal imitation). This article surveys evidence from developmental psychology, comparative neuroanatomy, and cognitive neuroscience indicating that human imitation and language are the result of brain adaptations shaped predominantly by cultural evolution. Rather than being an exclusively innate ability, the evidence reviewed points toward the idea of imitation as an ability that develops during infancy and childhood, supported by the maturation of sensorimotor brain networks and domain-general associative learning of multimodal information, both fostered by socially-rewarding interactions.

### What Is the Role of the Mirror Neuron System for Imitation and Communication?

Iacoboni and Dapretto (2006) proposed a neural circuit for imitation that includes the pSTS where visual input is processed and sent to the inferior parietal lobule, which is concerned with the motoric aspect of the action and projects into the IFG and ventral premotor cortex, where the goal of the action is recognized. Importantly, they also claim the existence of “efference copies of motor imitative commands that are sent back to the STS to allow matching between the sensory predictions of imitative motor plans and the visual description of the observed action” (Iacoboni and Dapretto, 2006). This network represents a suitable candidate to coordinate the processing of visual information and the execution of the corresponding motor sequence required for the imitation of facial expressions, such as lip or tongue protrusion. It is noteworthy that the areas involved in this circuit widely overlap with well-established regions of the mirror neuron system. The findings of the current review point toward a substantial role of the mirror properties of these brain areas to support the learning of multimodal association.

### Does the Third Visual Pathway in the Language-Dominant Hemisphere Play a Role for Audiovisual Integration of Speech?

As discussed in the third section of this review, recent evidence suggests that the pSTS is part of a third visual pathway that plays a critical role for social perception. Since it is specialized for the processing of biological movements in both human and NHPs, this area seems highly suited for gesture and facial expression imitation. In the left, language-dominant hemisphere, neural populations of the pSTS preferentially respond to both orofacial movements and vocalizations. For instance, regions that respond preferentially to mouths (vs eyes) also fire in response to conspecific voices. In line with the latter, the temporal voice area identified in both human and NHPs has a privileged location in the anterior temporal lobe, dorsally to STS. Although empirical studies are still needed to properly address this hypothesis, we suggest that the anatomical and functional characteristics of the third visual pathway in the left hemisphere turns it into a fitted circuit to support audiovisual integration of speech and lip-smacking. Future research in this field should investigate on brain activity lateralization during the processing of speaking faces in the other regions of the third visual pathway, namely, early visual areas (V1 and MT/V5) as well as the aSTS.

### What Can Cross-Species Homologies and Differences Tell Us About the Evolutionary Origins of Speech?

In the last section of this review, we offer insights about the phylogenetic evolution and ontological development of the multimodal integration of speech, accounting for cross-species homologies and differences in brain’s anatomy, function and developmental trajectories. We first reviewed evidence for a common evolutionary rhythm in humans and NHPs’ production of orofacial and vocal behaviors, phased-locked in the theta frequency-band with a peak around 4-to-5 Hz. It was suggested that this synchronization of visual (faces) and auditory (voices) cues during social communication emerges as a result of an intrinsic motor-speech rhythm imposed by a common generator, namely the vocal tract. Then, we surveyed humans and NHPs structural and functional homologies for the volitional control of the vocal tract in the vlPFC. Crucially, this region also responds to the perception of vocalizations and orofacial movements in both species, converting the vlPFC into a potential phylogenetically conserved trimodal region for the integration of audiovisual and motoric aspects of communication that may have contributed to the emergence of human speech (Aboitiz and García, 1997). Important cross-species differences have been documented, however, in the pattern of connectivity between LMC and brainstem nuclei. More specifically, the connections with those nuclei that control the muscles engaged in vocal folds vibrations and orofacial movements are more direct and robust in human brains compared to NHP brains. The strengthening of this structural connectivity across species evolution may have contributed to the development of finer vocal and orofacial motor control required for both imitation and speech production.

### What Do Species-Specific Sensory Development Can Tell Us About the Evolutive Origins of Speech?

We showed that despite some differences due to their neural development timing, humans and NHPs share similar developmental trajectories for multimodal integration of social stimuli. Noticeably, within their first year of life, infants of both species show a progressive attunement for the processing of native or species-specific visual (faces) and auditory (vocalizations) social stimuli. Importantly, this perceptual narrowing is highly influenced by environmental variables, such as enriched linguistic environment or the contingency of parental feedback, supporting the notion that early multimodal association learning is mediated by the engagement with socially relevant and rewarding interactions. In turn, since infants who dedicate greater attentional resources to the mouth (vs the eyes) of a speaker show greater expressive language development, we argue that the visual speech cues offered by speakers’ mouth movements are an important part of linguistic input during infancy and childhood, that benefits both language perception and production. Crucially, the prolonged wearing of opaque facemasks in nurseries and pre-school teachers in the context of current global pandemic may have adverse consequences for infants’ language acquisition, especially those with language learning impairments, since visual speech cues are no longer accessible in a speaker wearing an opaque mask. Finally, as mentioned at the end of the last section, human infants visually engage with the mouth to a greater extent than macaque infants do, suggesting an increased reliance on the information conveyed by orofacial movements is required for language acquisition relatively to lip-smacking, which involves less complex articulatory sequences and vocalizations than human speech.

## Author Contributions

MM took the lead in writing the manuscript. JZ-A and FA provided critical feedback and helped shape the theoretical analysis and the manuscript’s text. All authors contributed to the manuscript planification.

## Conflict of Interest

The authors declare that the research was conducted in the absence of any commercial or financial relationships that could be construed as a potential conflict of interest.

## Publisher’s Note

All claims expressed in this article are solely those of the authors and do not necessarily represent those of their affiliated organizations, or those of the publisher, the editors and the reviewers. Any product that may be evaluated in this article, or claim that may be made by its manufacturer, is not guaranteed or endorsed by the publisher.
